# Evaluation of care of dentoalveolar trauma

**DOI:** 10.1590/S1678-77572010000400004

**Published:** 2010

**Authors:** Luiz Fernando FARINIUK, Maria Helena de SOUSA, Vânia Portela Dietzel WESTPHALEN, Everdan CARNEIRO, Ulisses X SILVA NETO, Liliane ROSKAMP, Ana Égide CAVALI

**Affiliations:** 1 DDS, MSc, PhD Professor of Endodontics, Department of Dentistry, Pontifical Catholic University of Paraná, Curitiba, PR, Brazil.; 2 DDS, MSc, Professor of Endodontics, Department of Dentistry, Pontifical Catholic University of Paraná - PUCPR.; 3 DDS, MSc, Pontifical Catholic University of Paraná, Curitiba, PR, Brazil.

**Keywords:** Tooth injury, Dentoalveolar trauma, Examination, Prevalence

## Abstract

**Objectives:**

The aim of this study was to evaluate cases of dental trauma treated at the specialized center of Pontifical Catholic University of Paraná, Curitiba, Brazil, during a period of 2 years.

**Material and Methods:**

A total of 647 patients were evaluated and treated between 2003 and 2005. Data obtained from each patient were tabulated and analyzed as to gender, age, etiology, time elapsed after the injury, diagnosis (type of trauma), and affected teeth.

**Results:**

The results revealed that male individuals aged 7 to 13 years presented the highest prevalence of injury, and falling was the main causal factor. In most cases, the time elapsed between the accident and the first care ranged from 4 to 24 h. A total of 1,747 teeth were affected, with higher incidence of concussion/subluxation and coronal fracture, followed by lateral luxation and avulsion. The permanent maxillary central incisors were the most commonly affected teeth.

**Conclusion:**

The frequency and causes of dentoalveolar trauma should be investigated for identification of risk groups, treatment demands and costs in order to allow for the establishment of effective preventive measures that can reduce the treatment duration and costs for both patients and oral health services.

## INTRODUCTION

Dentoalveolar traumas are observed and treated in dental clinics. Their severity depends on the energy of impact and direction of the causal agent, as well as on the resistance of the tissues surrounding the traumatized teeth, which are more susceptible at the anterior region^11^, along with immunological factors, particularly in cases of avulsion and replantation^15^. Situations such as car, sports and working accidents, and falling are the most common reasons for dental traumatism^7,11,20^.

Facial and dental injuries have become an epidemiological health problem and may be more frequent than periodontal disease and caries in a near future, causing social, esthetic and psychological disturbances to the patients^3,14,18^. In the present study, an epidemiological evaluation of patients attending the Dentoalveolar Trauma Care Service of the Pontifical Catholic University of Paraná, Brazil, between 2003 and 2005, was undertaken to better analyze the requirements of emergency assistance.

## MATERIAL AND METHODS

Sixty hundred and forty seven patients with dentoalveolar trauma were treated at the Dentoalveolar Trauma Care Service of the Pontifical Catholic University of Paraná, Brazil, between 2003 and 2005. Informed consent was obtained from all patients for collection of data from their dental charts, and the study protocol was approved by the University Research ethics Committee (Protocol #1406). Information referring to gender, age, etiology, period of the year and hour of occurrence, time elapsed after the injury, diagnosis (type of trauma) and most affected teeth, were retrieved, plotted and presented in tables for further analysis.

## RESULTS

In the 3-year period of this study with a sample of 647 patients, dentoalveolar traumas occurred more frequently in males, accounting for 64.1% of cases, with mean age of 16.09 years. The most affected age range was 7 to 13 years (31.4%), followed by 14 to 21 years (23.6%) (Table 1). Analysis of the etiology of injuries revealed that falling, car accidents and physical aggression were the main causal factors (Table 2). With regard to the time elapsed after the injury until first care was provided, 32.6% of the subjected sought treatment within 4 to 24 h after injury, followed by 2 to 4 h (20.6%) (Table 3). A total of 1,747 teeth were affected. Most injuries were concussion/subluxation (23.8%), coronal fracture (23.7%), followed by lateral luxation (13.7%) and avulsion (13.6%) (Table 4). The permanent maxillary central incisors were the most affected teeth (53.2%), followed by the permanent maxillary lateral incisors (17.1%) and the primary maxillary central incisors (10.3%). The most frequent lesions in the permanent maxillary central incisors were coronal fracture (28.0%), concussion/subluxation (23.7%) and avulsion (15.2%) (Table 5).

**Tabela 1:** Age range gender divided into female and male

**Gender**			**Age range (percentage)**				**Total**
	**0-6**	**7-13**	**14-21**	**22-29**	**30-40**	**> 40**	
							
Female	56 (24.1)	82 (35.3)	35 (15.1)	34 (14.7)	19 (8.2)	6 (2.6)	232 (100)
Male	62 (14.9)	121 (29.2)	118 (28.4)	62 (14.9)	37 (8.9)	15 (3.6)	415 (100)
Total	118 (18.2)	203 (31.4)	153 (23.6)	96 (14.8)	56 (8.7)	21 (3.2)	647 (100)

**Tabela 2:** Etiology of dental trauma

**Etiology**	**N (%)**
	
Car accident	85 (13.10)
Sports accident	21 (3.20)
Physical aggression	74 (11.40)
Running over	26 (4.00)
Frontal crash	53 (8.20)
Falling	349 (53.90)
Others	23 (3.60)
Unreported	16 (2.50)
Total	647 (100.00)

**Tabela 3:** Time elapsed between the accident and the first care

**Time elapsed**	**N (%)**
	
<1 hour	110 (103)
1-2 h	223 (214)
2-4 h	197 (129)
>4 to 24 h	134 (121)
Up to 7 days	202 (210)
> 7 days	-
unreported	24 (20)
total	43 (53)

**Tabela 4:** Frequency of each diagnosis of dental trauma

**Diagnosis (type of trauma)**	**N (%)**
	
Avulsion	237 (13.6)
Concussion/subluxation	416 (23.8)
Extrusion	110 (6.3)
Crown fracture	414 (23.7)
Crown-root fracture	29 (1.7)
Bone fracture	85 (4.9)
Root fracture	104 (6.0)
Intrusion	113 (6.5)
Lateral luxation	239 (13.7)
Total	1,747 (100)

**Tabela 5:** Frequency of the different types of trauma to the permanent maxillary central incisors

**Diagnosis (type of trauma)**	**N (%)**
	
Avulsion	141 (15.2)
Concussion/subluxation	220 (23.7)
Extrusion	56 (6.0)
Crown fracture	260 (28.0)
Crown-root fracture	11 (1.2)
Bone fracture	38 (4.1)
Root fracture	74 (8.0)
Intrusion	42 (4.5)
Lateral luxation	87 (9.4)
Total	929 (100)

## DISCUSSION

An analysis of investigations of dentoalveolar traumas reveals that comparisons are very complex due to the different research methodologies employed^8^. The prevalence of dentoalveolar trauma varies according to the type of study, country where the study was conducted, and even different regions in a single country^7^. Statistics reveal that 4.2% to 36% of children, adolescents and young adults have already experienced dental trauma^7^.

In the present study, most traumas affected male individuals, as reported in other studies^13,19,20^. Boys are usually more susceptible to traumatic tooth injuries due to their greater involvement in sports activities, car accidents and fights^12^. The most frequently affected age range was 7 to 13 years, accounting for 31.4% of cases. Sakai, et al.^16^ (2005), found a higher incidence in children aged 0 and 3 years (34.42%), followed by those in the 7-12-year-old group (18.12%). The most frequent etiologic agents were falls, car accidents and physical assaults, which agree with the findings of other studies^2,7,17^.

According to the diagnosis (type of trauma), there was higher incidence of concussion/subluxation, followed by coronal fracture, lateral luxation and avulsion. Some studies found different results^4,5^, yet others agree with the present findings^12,20^. The high rate of luxation and avulsion were probably related to the severity of injuries.

As far as the time elapsed after the trauma until first care was provided, 74.4% of patients seen by a dentist in the same day of the accident, in most after 4 h. Only 5.9% of the cases were treated up to 1 h after the injury, which is probably due to the fact that dentists are not always the first health professionals assisting these patients, who often search for care at hospital emergency units. It has also been observed that decisions taken by health professionals, including dentists, are not always correct, which delays proper care and impairs the prognosis in medium and long term, due to the lack of knowledge of the management of dental trauma^5,10^.

Immediate care is required in cases of dentoalveolar trauma. This type of emergency situation often requires several sessions for treatment, continuity for investigation and even treatment of possible sequelae^1,6^.

The most affected teeth were the permanent maxillary central incisors, accounting for 53.2% of cases, which exhibited higher occurrence of coronal fracture, concussion/subluxation, and avulsion.

Some epidemiological studies are conducted at hospitals^2^, whereas others are conducted at Pediatric Dentistry clinics^9^. The present study was conducted at a specialized facility that treats only patients with dental trauma. This dentoalveolar trauma care service was created due to the gap existing in this type of care, especially concerning healthcare to the poor population.

## CONCLUSION

The frequency and causes of dentoalveolar trauma should be investigated for identification of risk groups, treatment demands and costs in order to allow for the establishment of effective preventive measures that can reduce the treatment duration and costs for both patients and oral health services. educational campaigns are needed in order to inform teachers, parents and health professionals about the best emergency measures, and reduce the time elapsed between the dental trauma and the first care.
